# CHIASM, the human brain albinism and achiasma MRI dataset

**DOI:** 10.1038/s41597-021-01080-w

**Published:** 2021-11-26

**Authors:** Robert J. Puzniak, Brent McPherson, Khazar Ahmadi, Anne Herbik, Jörn Kaufmann, Thomas Liebe, Andre Gouws, Antony B. Morland, Irene Gottlob, Michael B. Hoffmann, Franco Pestilli

**Affiliations:** 1 Visual Processing Lab, Department of Ophthalmology, Otto-von-Guericke-University, Leipziger-Str. 44 (H. 60B), 39120 Magdeburg, Germany; 2 Pestilli Lab, Department of Psychological and Brain Sciences, Indiana University Bloomington, 1101 E 10th Street, Bloomington, Indiana 47405 USA; 3 Department of Neurology, Otto-von-Guericke-Universität, Leipziger-Str. 44 (H. 60A/60B), 39120 Magdeburg, Germany; 4 Department of Psychiatry and Psychotherapy, Jena University Hospital, Philosophenweg 3, 07742 Jena, Germany; 5 York Neuroimaging Centre, Department of Psychology, University of York, York, YO10 5DD United Kingdom; 6 Centre for Neuroscience, Hull-York Medical School, Heslington, York, YO10 5DD United Kingdom; 7 Department of Neuroscience, Psychology & Behaviour, University of Leicester, University Road, Leicester, LE1 7RH United Kingdom; 8 Center for Behavioral Brain Sciences, Otto-von-Guericke-Universität, Universitätsplatz 2 (G24-205), 39106 Magdeburg, Germany; 9 Department of Psychology, Center for Perceptual Systems, Center for Theoretical and Computational Neuroscience, Institute for Neuroscience, The University of Texas, 108 E Dean Keeton Street, Austin, Texas 78712 United States

**Keywords:** Visual system, Biophysical models, Neurodevelopmental disorders, Visual system, Axon and dendritic guidance

## Abstract

We describe a collection of T1-, diffusion- and functional T2*-weighted magnetic resonance imaging data from human individuals with albinism and achiasma. This repository can be used as a test-bed to develop and validate tractography methods like diffusion-signal modeling and fiber tracking as well as to investigate the properties of the human visual system in individuals with congenital abnormalities. The MRI data is provided together with tools and files allowing for its preprocessing and analysis, along with the data derivatives such as manually curated masks and regions of interest for performing tractography.

## Abstract (summary)

**Table:** 

Measurement(s)	brain white matter
Technology Type(s)	magnetic resonance imaging
Sample Characteristic - Organism	Homo sapiens

Machine-accessible metadata file describing the reported data: 10.6084/m9.figshare.16726246

## Background & Summary

We present CHIASM, the human brain albinism, and achiasma dataset, a unique collection of magnetic resonance imaging (MRI) data of brains with congenital abnormalities in the visual system. The unique feature of these subjects is the varied amount of crossing found in a specific structure –the optic chiasm– across participants with albinism. More specifically, it is well established^1^ that the number of crossing fibers crossing at the human optic chiasma to reach the contralateral brain hemisphere (right and left respectively) varies between certain groups. The percentage of fiber crossing at the chiasm has been reported for normal-sighted (control) participants to be about 53%^2^. In brains affected by albinism instead, the number of crossing fibers at the optic chiasm grows above 53%^3^. Crossing fibers within the optic chiasma in individuals affected by chiasm hypoplasia is lower than 53%^4^. Finally, data from individuals with achiasma have been shown to completely lack neuronal fiber crossing at the optic chiasm^1,5,6^.

**Fig. 1:**
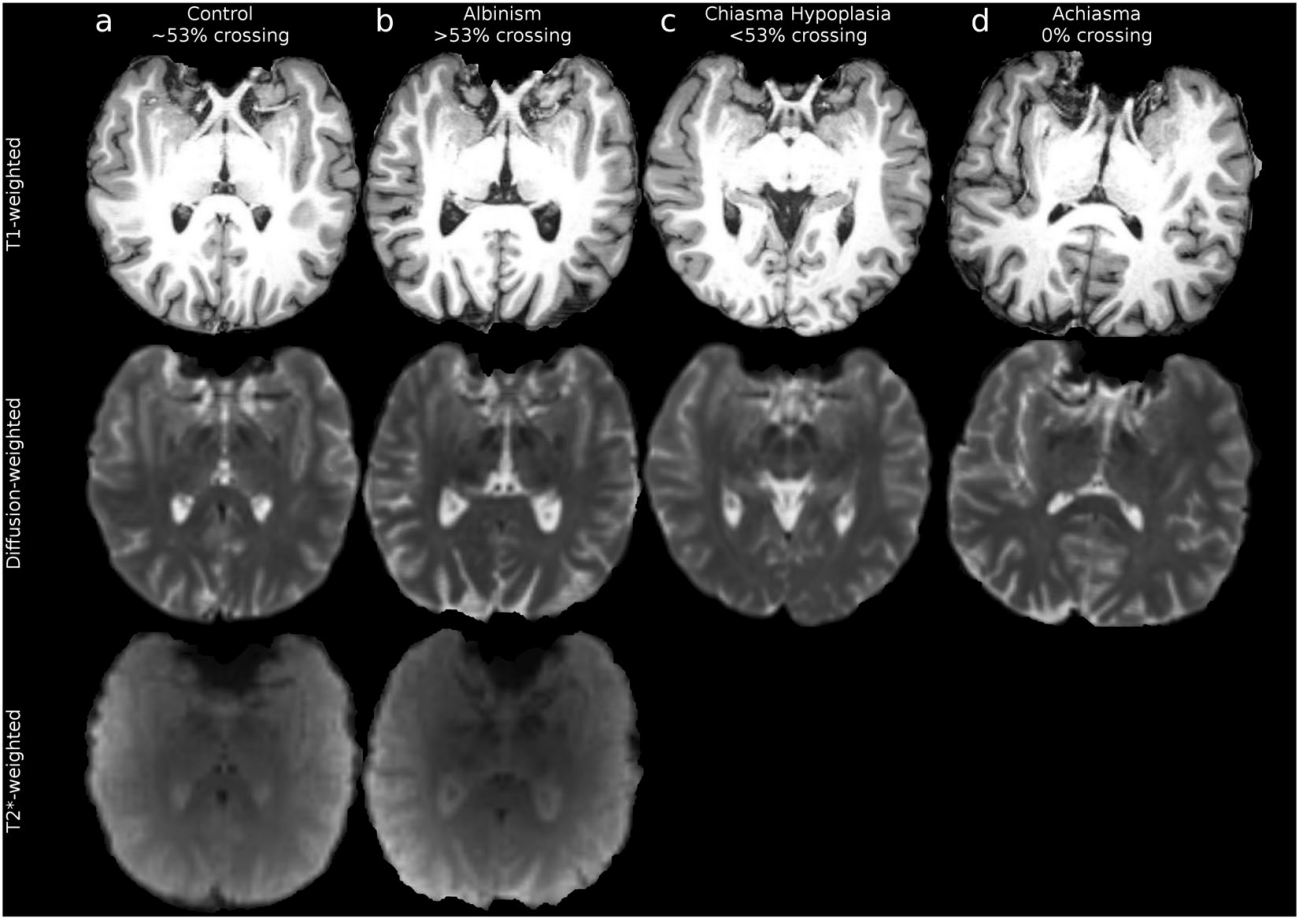
Overview of structural abnormalities of the optic chiasm and provided MRI data. (**a**) Exemplary control participant (CON1). (**b**) Exemplary participant with albinism (ALB1). (**c**) Participant with chiasma hypoplasia (CHP1). (**d**) Participant with achiasma (ACH1). The fMRI data is not provided due to severe nystagmus and motion compromising the quality of data. Top, middle and bottom rows display respectively T1w, DW, and fMRI data. Images show pseudo-axial views of a T1w image cropped to the brain mask.

The data covers four participant groups: the controls (n = 8, Fig. 1a), albinism (n = 9, Fig. 1b), chiasma hypoplasia (n = 1; Fig. 1c) and achiasma (n = 1; Fig. 1d), and comprises three different MRI modalities: (A) T1-weighted (T1w; Fig. 1, top row) images, (B) diffusion-weighted images (DWI, Fig. 1, middle row) and (c) T2*-weighted functional MRI (fMRI; Fig. 1, bottom row) images. More specifically: (A) T1w images are provided together with further derivatives (masks and labels obtained through segmentation, white matter mask manually curated in optic chiasm region), (B) DWI data was acquired using high angular^7,8^ and spatial^9–11^ resolution and is provided with further derivatives, such as tractography results, (C) fMRI data is provided for the subset of participants from control and albinism groups (n = 4 and n = 6, respectively) and is accompanied with meta-files describing stimulus and acquisition. All the data, both in the raw and preprocessed form are available on the cloud computing platform https://brainlife.io^11^ and Github repository https://github.com/rjpuzniak/CHIASM.

The data we present here can be of value to the scientific community for multiple reasons. First, it can serve as a reference dataset to support basic research for clarifying the neuroscientific underpinnings of the different conditions. Currently, there are no reference datasets available covering similar conditions measured with high-resolution DWI data. Second, this dataset can be used by investigators to validate independent results and advance studies on the disease and neuroplasticity mechanisms. This is possible as chiasmal malformations induce abnormal representations within the visual pathways, which are expected to trigger neuroplastic mechanisms e.g. to resolve potential sensory conflicts^1,12–19^. Finally, the dataset presented here can be used to advance tractography methods development. The field of brain tractography has faced a long-lasting challenge commonly referred to as the “crossing fibers problem” or simply CFP^18,20–35^. CFP can lead to poor estimates of the number of crossing fibers through brain regions containing multiple fiber populations^36,37^. It has been established that up to 90% of total brain white matter volume might have crossing fibers^38^. Advancing methods for accurate tracking in regions with crossing fibers is fundamental in clarifying the role of white matter in human health and disease^18,39,40^. As of today, several important approaches to tractography evaluation and validation have been proposed. These approaches can be classified into four primary categories: synthetic phantoms^41,42^, physical phantoms^43^, biological phantoms^44,45^, and statistical^9,32,46–49^. Most of these approaches have helped advance tractography methods, but major challenges remain^30,31,42^. The data made available here opens the possibility to assess crossing strength at the optic chiasm by first using anatomical data (T1w, DWI) to model the crossing at the optic chiasm and cross-validating the proposed findings with functional estimates (fMRI) of misrouting based on the BOLD signal^4,50,51^ (see Suppl. Table 1). This provides a unique opportunity for testing novel tractography methods that assess crossing strength by providing an independent modality for their evaluation.

## Methods

### MRI Data sources

The described MRI data was analyzed in previously published studies^4,50,51^, where acquisition protocols and data properties are detailed.

### Participants

A single participant with achiasma, a single participant with chiasm hypoplasia, 9 participants with diagnosed albinism, and 8 control participants [no neurological or ophthalmological history; normal visual acuity (≥1.0 with Freiburg Visual Acuity Test^52^) and normal stereo vision^53,54^] were recruited for the MRI measurements. Each participant was instructed about the purpose of the study and the methods involved and gave written informed study participation and data sharing consent. The study was approved by the Ethics Committee of the Otto-von-Guericke University Magdeburg, Magdeburg, Germany. The patients and control participants underwent ophthalmological examination (Suppl. Table 1), which incorporated methods described in^55,56^.

### MRI Data acquisition

MRI data was acquired with a Siemens MAGNETOM Prisma 3 Tesla scanner with the *Syngo* MR D13D software and a 64-channel head coil. The acquisition protocol for T1w and DW data was initiated by a localizer scan, followed by a whole-brain T1w 3D-MPRAGE scan and two DW scans - respectively with anterior-posterior (A-P) and posterior-anterior (P-A) phase-encoding direction. T1w and DW images were collected during a single continuous scanning session, fMRI data was acquired in separate sessions (patients data was acquired on two consecutive days). T1w images were obtained in sagittal orientation using a 3D-MPRAGE sequence (TE/TR = 4.46/2600 ms, TI = 1100 ms, flip angle = 7°, resolution = 0.9 × 0.9 × 0.9 mm^3^, FoV: 230 × 230 mm²; image matrix: 256 × 256 × 176, acquisition time = 11 min:06 s^57^) and corrected simultaneously during acquisition for gradient nonlinearity distortions. Each individual’s T1w data was screened by a radiologist for unexpected abnormalities present in the data. Apart from the abnormalities given in Methods (Participants), no clinically relevant abnormalities were detected.

DWI were acquired with Echo-Planar Imaging (EPI) sequence (TE/TR = 64.0/9400 ms, b-value 1600 s/mm², resolution 1.5 × 1.5 × 1.5 mm³, FoV 220 × 220 mm², anterior to posterior (A-P) phase-encoding direction, acquisition time = 22 min:24 s, no multi-band). The b-value was chosen with regard to reported optimal values for resolving two-way crossing^58^ (1500–2500 s/mm^2^). Scans were performed with 128 gradient directions, so the obtained DWI data can be described as High Angular Resolution Diffusion Imaging^7^ (HARDI) data. The redundantly high number of gradient directions for the maximal angular contrast provided by a b-value of 1600 s/mm^2^ supported residual bootstrapping. This enhanced the effective signal-to-noise ratio (SNR), which is an important feature considering the reduced SNR of the DWI of the optic chiasm. The gradient scheme, initially generated using E. Caruyer’s tool for q-space sampling^59^ for 3 shells acquisition, was narrowed to the single shell in order to address the acquisition time constraints. DW volumes were evenly intersected by 10 non-diffusion weighted (b-value = 0, hereafter referred to as b0) volumes for the purpose of motion correction. The second DW series were acquired with reversed phase-encoding direction in comparison to the previous scan, specifically posterior to anterior (P-A). Apart from that, all scan parameters were identical to ones corresponding to the preceding acquisition. Acquisition of two DW series with opposite phase-encoding directions enhanced the correction of geometrically induced distortions^60^. Furthermore, the additional scans improve the signal-to-noise ratio (SNR) of the total DWI data.

fMRI data was acquired from 4 controls and 6 participants with albinism (see Suppl. Tables 1 and 2) with T2*-weighted EPI sequence (TE/TR = 30.0/1500 ms, flip angle = 70°, resolution 2.5 × 2.5 × 2.5 mm³, FoV 210 × 210 mm^2^, acquired with multi-band and in-plane acceleration factor = 2) during visual stimulation. Visual stimulation was performed in either the left, right or both visual hemifields in separate runs. A single repetition comprised 168 volumes acquired within 252 seconds. Each of these three stimulation conditions was repeated three times, resulting in a total of nine functional runs acquired within a single session. The visual stimulation is detailed in^51^. Briefly, it employed a moving high-contrast checkerboard pattern^61^ presented within the aperture of a drifting bar (width: 2.5°) within a circular aperture (radius: 10°). The bar aperture was moving in four directions (upwards, downwards, left, and right) across the stimulus window in 20 evenly spaced steps within 30 s. The sequence of the visual stimulation runs was interspersed by equally long (30 s) mean luminance blocks with zero contrast. The stimuli, generated with Psychtoolbox^62,63^ in MATLAB (Mathworks, Natick, MA, USA), were projected onto a screen (resolution 1140 × 780 pixels) placed at the magnet bore. The participants viewed the stimuli monocularly with their dominant eye (see Suppl. Tables 1 and 2) via an angled mirror at a distance of 35 centimeters, and were instructed to fixate on a central dot and respond with a button press to dot color changes.

#### Data preprocessing

Data preprocessing was mainly performed online, using web services available on the brainlife.io platform (https://brainlife.io), with a few exceptional steps done offline. The source code for the Apps used for online preprocessing is to be found at https://github.com/brainlife. The offline preprocessing involved conversion of DICOM data to NIfTI format, data anonymization, and, in the case of DW data, correction of gradient nonlinearity distortions and alignment to T1w image. The scripts for all of the offline preprocessing steps are available on https://github.com/rjpuzniak/CHIASM. Data preprocessing was meant to provide minimally processed data and standardized T1w, DWI, and fMRI data files.

The following software packages were used for data preprocessing: MRtrix^28,64^, FMRIB’s FSL^65–67^, ANTs^68,69^, FreeSurfer^70^, dcm2niix^71^, MIPAV^72^, VISTASOFT package (including mrVista and mrDiffusion tools; https://github.com/vistalab/vistasoft), AFNI^73^, fMRIPrep^74^, Mindboogle^75^, Nipype^76^, Nilearn^77^ and Human Connectome Project gradunwarp package (https://github.com/Washington-University/gradunwarp). The computing environment of the brainlife.io uses Docker (https://docker.com) as well as Singularity containers (https://sylabs.io/singularity/ and https://singularity.lbl.gov).

### Preprocessing of the T1w data

**Fig. 2:**
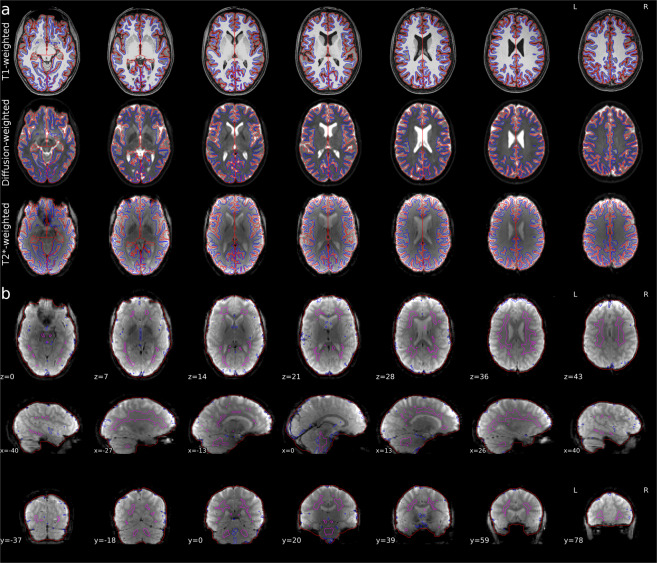
Qualitative overview of preprocessing for a representative participant (CON1). (**a**) Axial view of surfaces of white and pial matter (blue and red color, respectively) overlaid on T1w (top row), non-diffusion weighted (b0; middle row) and fMRI (T2*; bottom row) images. (**b**) Axial (top row), sagittal (middle row) and coronal (bottom row) views of fMRI images. The red contour marks brain mask estimated from BOLD signal, magenta contour marks combined CSD and WM masks, where voxels with partial GM volume were removed, blue contour marks the top 2% most variable voxels within brain mask.

In the offline preprocessing steps, T1w images were converted into the NIfTI format using *dcm2niix*^71^ and subsequently anonymized through the removal of facial features using *mri_deface* algorithm^78^ from FreeSurfer 6.0.0. Anonymized T1w images were aligned to the Anterior Commissure - Posterior Commissure (ACPC) plane using the mrAnatAverageAcPcNifti.m command from mrDiffusion package (https://github.com/vistalab/vistasoft/wiki/ACPC-alignment). The outcome T1w images were used as the reference image for the coregistration of DWI. Further, T1w images were automatically segmented into five-tissue-type (cerebrospinal fluid, white, grey, and subcortical grey matter, and eventual pathological tissue; 5TT) segmented images^79^ through the use of commands from FSL 6.0.3^65,80–82^. Finally, the T1w data was uploaded to brainlife.io, where it was segmented once again using FreeSurfer 7.1.1 (Fig. 2a, top row). Detailed information about the preprocessing code is provided in the Code Availability section (Table 1).

**Table 1:** List of preprocessing steps applied to the T1w images, together with web links to relevant software and, if available, brainlife.io Apps.

Preprocessing step	Software/Tool	Software website/App
1. DICOM conversion	dcm2niix	http://people.cas.sc.edu/rorden/mricron/dcm2nii.html
2. Anonymization	FreeSurfer 6.0.0/mri_deface	https://surfer.nmr.mgh.harvard.edu/fswiki/mri_deface
3. ACPC Alignment	mrDiffusion/mrAnatAverageAcPcNifti	https://github.com/vistalab/vistasoft
4. Tissue Segmentation	MRtrix 3.0/5ttgen	https://www.mrtrix.org
5. Tissue segmentation	FreeSurfer 7.1.1/recon-all	https://surfer.nmr.mgh.harvard.edu
		10.25663/brainlife.app.462^124^

Web services used to process that are available for reuse on https://brainlife.io/apps.

### Preprocessing of the DWI data

The DICOM DWI data preprocessing followed the well-established outline proposed by the Human Connectome Project (HCP) consortium^83^. Initially, DW files were converted offline into NIfTI format using *dcm2niix* and uploaded to brainlife.io. Next, the DWI data was corrected online for the Rician noise using *dwidenoise*^84,85^ and for Gibbs ringing using *mrdegibbs*^86^ commands from MRtrix 3.0. The following step of preprocessing involved estimation of the susceptibility-induced off-resonance field in the DW data with FSL’s *topup* command^60,65^ using two DW series with opposite phase encoding directions. The output of *topup* was subsequently fed to *eddy* command^87^ in order to correct for susceptibility- and eddy current-induced off-resonance field, as well as the motion correction. The *topup* and *eddy* command were implemented through *dwifslpreproc* command from MRtrix 3.0, which final output was a single file containing the corrected DW series. In the final online preprocessing step, the data was corrected for the field biases using *dwibiascorrect* from MRtrix 3.0, which, in turn, used the N4 algorithm from ANTS^88^ in order to estimate the MR field inhomogeneity. At this stage, the DWI data were downloaded and corrected in an offline mode for the gradient nonlinearities distortions. This step involved using the *gradunwarp* package and information about Legendre coefficients in spherical harmonics for the scanner’s gradient coil, provided by the vendor (stored in the https://github.com/rjpuzniak/CHIASM repository). As for the final step of preprocessing, the DWI data was coregistered to T1w data using the Boundary-Based Registration (Fig. 2a, middle row). At first, the transformation matrix from DWI to T1w image space was estimated with the *epi_reg* command from FLIRT^89–91^, a part of FSL 6.0.3 package. The transformation matrix was subsequently applied to DWI data by the *flirt* command from the same package, and to the corresponding b-vectors by shell script from HCP repository (https://github.com/Washington-University/HCPpipelines/blob/master/global/scripts/Rotate_bvecs.sh). The resulting data, in NIfTI format, have been uploaded to brainlife.io and were published as a preprocessed DW data set. Detailed information about the preprocessing code is provided in the Code Availability section (Table 2).

**Table 2:** List of preprocessing steps applied to the DW images, together with web links to relevant software and, if available, brainlife.io Apps.

Preprocessing step	Software/Tool	Software website/App
1. DICOM conversion	dcm2niix	http://people.cas.sc.edu/rorden/mricron/dcm2nii.html
2. Denoising	MRtrix 3.0/dwidenoise	https://www.mrtrix.org
		10.25663/bl.app.68125
3. Removal of Gibbs ringing	MRtrix 3.0/mrdegibbs	https://www.mrtrix.org
		10.25663/bl.app.68^125^
4. Geometrical distortions corrections	FSL/topup	https://fsl.fmrib.ox.ac.uk/fsl/fslwiki
		10.25663/bl.app.68^125^
5. Eddy currents distortions corrections	FSL / eddy	https://fsl.fmrib.ox.ac.uk/fsl/fslwiki
		10.25663/bl.app.68^125^
6. Correction for head motion	FSL/eddy	https://fsl.fmrib.ox.ac.uk/fsl/fslwiki
		10.25663/bl.app.68^125^
7. Correction for bias field	MRtrix 3.0/dwibiascorrect	https://www.mrtrix.org
		10.25663/bl.app.68^125^
8. Correction for gradient nonlinearities	gradunwarp/gradunwarp	https://github.com/Washington-University/gradunwarp
9. Coregistration to T1w image & Rotation of b-vectors	FSL/epi_reg & flirt MRtrix/mrresize HCP Pipelines/rotate_bvecs	https://fsl.fmrib.ox.ac.uk/fsl/fslwiki
		https://www.mrtrix.org
		https://github.com/Washington-University/HCPpipelines/blob/master/global/scripts/Rotate_bvecs.sh

Web services used to process that are available for reuse on https://brainlife.io/apps.

### Preprocessing of the fMRI data

The fMRI data was converted into NIfTI format using dcm2niix. Subsequent preprocessing was performed online using two Apps wrapping the fMRIPrep tool^74^: *fMRIPrep - Surface output*^92^ (which output data as the surface vertices) and *fMRIPrep - Volume output*^93^ (which output data in volumetric format). The preprocessing, in both cases, involved correction for susceptibility distortions using *antsRegistration* from ANTs 2.3.3, registration to T1w image using *bbregister* command from FreeSurfer 6.0.1 (Fig. 2a, bottom row), slice-time correction using *3dTshift* from AFNI and correction for head-motion. Additionally, the BOLD (blood-oxygen-level-dependent) data was subject to Component-Based Noise Correction (CompCor)^94^, which uses information principal components from noise-driven regions (defined as top 2% variable voxels in BOLD image; Fig. 2b, blue contour) in order to reduce the standard deviation of resting-state BOLD data. The noise-driven regions selection was limited only to voxels not affected by gray matter partial volume (Fig. 2b, pink contour). The output files created during preprocessing with *fMRIPrep* Apps are described in detail in section Data Records. Detailed information about the preprocessing pipeline is provided in the HTML report files generated by fMRIPrep application in the Data Records, while the code is provided in the Code Availability section (Table 3).

**Table 3:** List of preprocessing steps applied to the fMRI images, together with web links to relevant software and, if available, brainlife.io Apps.

Preprocessing step	Software/Tool	Software website/App
1. DICOM conversion	MRtrix 3.0/mrconvert	https://www.mrtrix.org
2. Geometrical distortions corrections	ANTS 2.3.3/antsRegistration	http://stnava.github.io/ANTs/
		10.25663/brainlife.app.160^93^.
		10.25663/brainlife.app.267^92^
3. Registration to T1w image	FreeSurfer 7.1.1/bbregister	https://surfer.nmr.mgh.harvard.edu/.
		10.25663/brainlife.app.160^93^
		10.25663/brainlife.app.267^92^
4. Slice-time correction	AFNI/3dTshift	https://afni.nimh.nih.gov/
		10.25663/brainlife.app.160^93^
		10.25663/brainlife.app.267^92^
5. Motion correction	FSL 5.0.9/mcflirt	https://fsl.fmrib.ox.ac.uk/fsl/fslwiki
		10.25663/brainlife.app.160^93^
		10.25663/brainlife.app.267^92^
6. Removal of physiological noise	CompCor	10.25663/brainlife.app.160^93^
		10.25663/brainlife.app.267^92^

Web services used to process that are available for reuse on https://brainlife.io/apps.

### Drawing of the optic chiasm mask

**Fig. 3:**
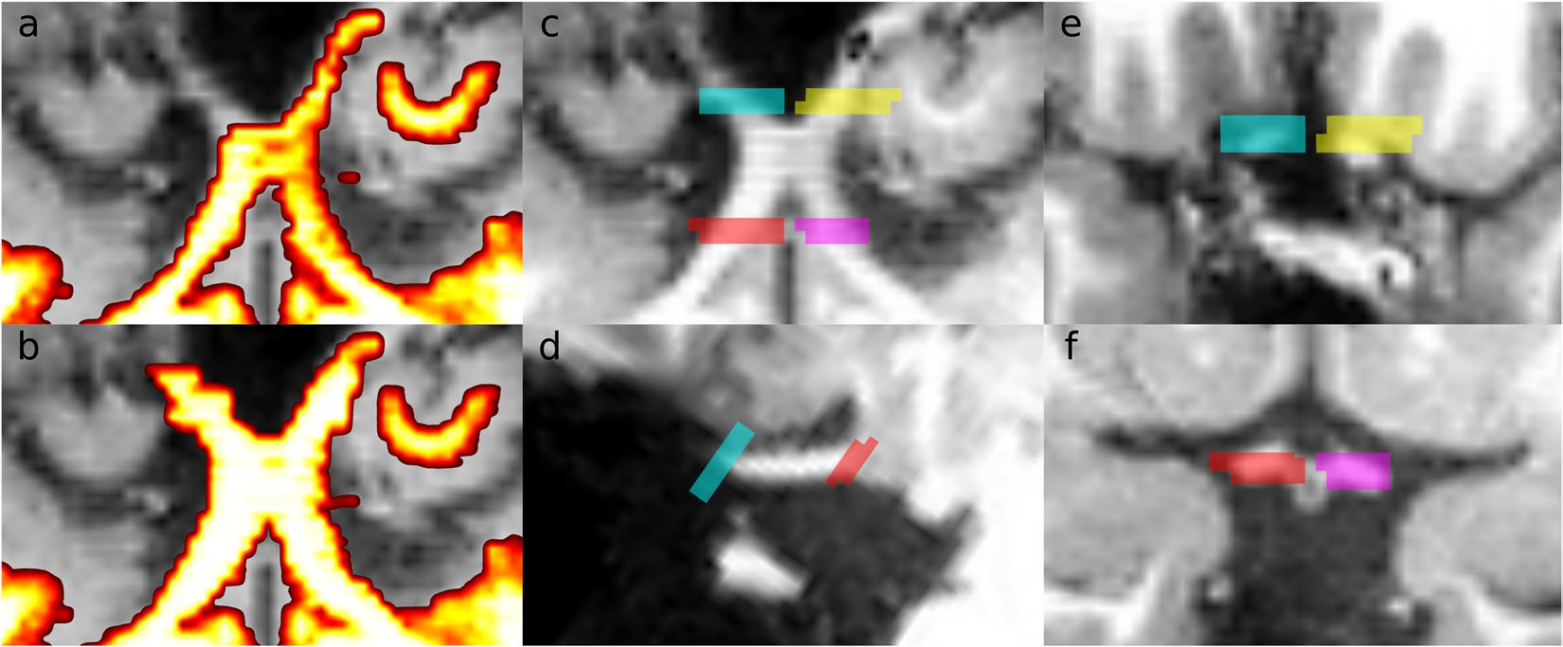
Masks and regions of interest used to define the location of the optic chiasm, tract, and nerve. Left-hand column, the pseudo-axial view of the anatomical image of exemplary control participant (CON6) and overlaid optic-chiasm white matter mask before (**a**) and after (**b**) manual correction. Middle column, four representative ROIs covering the optic nerves and optic tracts in pseudo-axial (**c**) and sagittal (**d**) view. Right-hand column, four representative ROIs covering a cross-section of optic nerves (**e**) and optic tracts (**f**) in pseudo-coronal view.

Due to the limited accuracy of the automatically generated optic chiasm mask (Fig. 3a), manual segmentation was necessary to ensure the proper anatomical definition of the structures in each participant. The procedure comprised the following steps:

1. Initial segmentation of voxels unambiguously belonging to the optic chiasm (i.e. outer voxels affected by partial volume effects were excluded). This segmentation was performed only in an axial view and was done in multiple slices covering optic nerves, optic chiasm,and optic tract.
2. Second step where voxels affected by partial volume effects, previously omitted, were included. The two main criteria for the inclusion of candidate voxels were (a) relative intensity (compared to neighboring voxels identified in the previous step) and the coherence/continuity of the optic chiasm structure (already defined by voxels selected in the previous step).
3. A third and final step involved corrections performed in axial, coronal, and sagittal views at the same time. The main criterion here was to assure the continuous borders.

The outcome masks covered posterior optic nerves, whole optic chiasm, and anterior optic tracts (Fig. 3b) and were used for correction of white matter definition in previously generated 5TT masks. The corrected white matter masks were extracted from 5TT images using *mrconvert* command, transformed to the space of T1w image (in order to ensure matching of QForm and SForm transformation matrices) with *flirt* command from FSL and *mrconvert* commands from MRtrix, and uploaded to the brainlife.io. Detailed information about the code for preprocessing can be found in the Code Availability section (Table 4).

**Table 4:** List of preprocessing steps applied to the custom white matter masks and ROIS, together with web links to relevant software and, if available, brainlife.io Apps.

Preprocessing step	Software/Tool	Software source/App
1. Alignment of the custom mask to T1w image	FSL/flirt	https://www.mrtrix.org
2. Alignment of the ROIs to T1w image	MRtrix/mrtransform	https://www.mrtrix.org
3. Removal of interpolation artifacts	MRtrix/mrthreshold	https://www.mrtrix.org

Web services used to process that are available for reuse on https://brainlife.io/apps.

### Drawing regions of interest in the optic chiasm

Four ROIs were manually drawn and curated in each participant (Fig. 3c) on the T1w images. These ROIs identified the anterior and posterior aspects of the optic chiasm in each individual. The two anterior ROIs identified the location of the left and right optic nerve (Fig. 3c,e,yellow, and magenta). The posterior ROIs identified the left and right optic tract (Fig. 3c,f, cyan, and red). Once created, ROIs were transformed to the space of T1w image with *mrconvert* and *mrtransform* and thresholded with *mrthreshold* commands from MRtrix (in order to remove interpolation artifacts) and uploaded to the brainlife.io. Detailed information about the code for preprocessing can be found in the Code Availability section (Table 4).

### Diffusion signal reconstruction, tractography, and statistical evaluation

The whole-brain tractography was performed using MRtrix 0.2.12^64^. The tractography was based on diffusion tensor (DT) and constrained spherical deconvolution (CSD) models and was performed using both deterministic and probabilistic methods^25,28,95–99^. The DT model was used for deterministic tracking, in the case of CSD both deterministic and probabilistic tracking was applied. The tractography utilized Anatomically-Constrained Tractography^79^, where the tracking was restricted to gray matter-white matter boundary from the newly created FreeSurfer 7.1.1 segmentation of the provided T1w image to ensure the agreement of the obtained tracks with the underlying anatomical structure. Additionally, an Ensemble Tractography^100^ framework was used, which negates bias of different parameter options in tractography by merging results of several tracking approaches, with each generating tracks with different properties^18,41,101–104^. Merging all tractograms mitigates the bias introduced from the variability of individual tractography parameters. Adhering to this, the final tractogram consisted of streamlines generated using: (A) deterministic tracking^97^ based on Diffusion Tensor^95,105^, (B) deterministic tracking^25^ based on Constrained Spherical Deconvolution model^106^ (CSD), and (C) probabilistic CSD-based tracking using iFOD2 algorithm^28^. The tractography was performed for several harmonic orders L = 2, 4, 6, 8, 10, 12, which were estimated using *dwi2reponse tournier*^107^ and *dwi2fod msmt_csd*^108^ commands from MRtrix 3.0. The full set of parameters guiding tractography is as follows: step size 0.15 mm for (A) and (B), 0.75 mm for (C); minimal length 7.5 mm, maximal length 200 mm, maximum angle between consecutive steps 5, 10, 20, 40, 80°, 15,000 fibers per parameters combination. The tracking has been performed using *Ensemble Tracking (dwi)*^109^ brainlife.io application.

The whole-brain tractogram was evaluated and optimized using the Linear Fasiscle Evaluation method^9,49^ (LiFE). Over the course of the evaluation process, every streamline is assigned a weight indicating its unique contribution in explaining the measured diffusion signal based on a tensor fit of the preprocessed diffusion data. Streamlines with non-zero weights are deemed as significant, while others are being discarded. The brainlife.io application implementing LiFE evaluation^9^ can be found at^110^.

## Data Records

The data includes T1w, DW, and (if available) fMRI images of: single participant with achiasma (ACH1), single participant with chiasma hypoplasia (CHP1), 9 participants with albinism (ALB1 - ALB9) and 8 control participants (CON1 - CON8). The data from control participants are provided under an open license. To assure anonymity of the participants with clinical conditions, their data are made available upon direct request (as regulated by the Data Use Agreement, Suppl. Box 1).

The data is publicly accessible via brainlife.io platform^11^ at 10.25663/brainlife.pub.9^111^. When downloaded, the files are organized as defined by brainlife.io DataTypes (https://brainlife.io/docs/user/datatypes/ and https://brainlife.io/datatypes), and, if applicable, as the most updated version of the Brain Imaging Data Structure specification^112^ (BIDS). Due to the developmental nature of the BIDS format, at the present time, it does not support all the data derivative types presented here; the data records detailed below are presented according to brainlife.io Data Types. The data files stored for each subject on brainlife.io can be divided into three general categories: (A) source data, which consist of anonymized and aligned to the anterior commissure - posterior commissure (AC-PC) space T1w image, raw DW and fMRI data in NIfTI format, (B) preprocessed data, which consist of preprocessed DW and fMRI data, as described in Data Preprocessing section and (C) data derivatives, as described in Data Derivatives section. Additionally, the fMRI NIfTI data stored on brainlife.io are provided together with (D) MrVista.mat files (further referred to as “fMRI meta-files”), which are necessary for the analysis of the former. Those files are stored in a separate Open Science Framework (OSF) repository: 10.17605/osf.io/XZ29Q^113^ and are described in detail in the ‘fMRI meta-files’ section.

### Source data

Source data (raw) files consist of two DWI datasets, one T1w set per participant, and, in the case of 6 participants from the albinism group and 4 controls, fMRI T2*-weighted images.

### DW source data

Source DWI data covers two DW series acquired with opposite phase encoding directions (PEDs) - Anterior-Posterior (AP, Box 1a) and Posterior-Anterior (PA, Box 1b), as indicated by the tags.

Box 1 Organization of the DW source data files according to the brainlife.io Data Types. (**a**) DW data files corresponding to acquisition with AP PED, (**b**) DW data files corresponding to acquisition with PA PED.

- proj-5ddfa986936ca339b1c5f455/sub-{}/dt-neuro-dwi.tag-raw.tag-AP.tag-normalized.tag-single_shell.id-{}/       dwi.bvals       dwi.bvecs       dwi.nii.gz       _info.json
- proj-5ddfa986936ca339b1c5f455/sub-{}/dt-neuro-dwi.tag-raw.tag-PA.tag-normalized.tag-single_shell.id-{}/dwi.bvals

      dwi.bvecs      dwi.nii.gz      _info.json

### fMRI source data

The source fMRI data is available for 6 participants with albinism (ALB1, ALB5, ALB6, ALB7, ALB8 and ALB9) and 4 controls (CON1, CON2, CON3 and CON8) and incorporates BOLD series acquired in 6 runs (3 runs corresponding to monocular stimulation of right visual hemifield, and 3 runs for left), except for ALB5 (3 runs for right and 2 for left hemifield) and CON1 (2 runs for right and 3 for left hemifield). Importantly, the fMRI files for the participant are stored in separate sessions (e.g. CON1/run4, Box 2).

Box 2 Organization of the fMRI source data files according to the brainlife.io Data Types. proj-5ddfa986936ca339b1c5f455/sub-{}.ses-run{}/dt-neuro-func-task.tag-raw.tag-retinotopy.id-{}/     bold.nii.gz     _info.json

### T1w source data

Source T1w images (already anonymized and aligned to ACPC plane) were uploaded to participant’s main folder (e.g. CON1/, Box 3a), and, if applicable, all fMRI sessions (e.g. CON1/run4, CON1/run5 etc., Box 3b).

Box 3 Organization of the T1w source data files according to the brainlife.io Data Types. (**a**) T1w images uploaded to participant’s main folder, (**b**) T1w images uploaded to all folders corresponding to separate fMRI sessions.

- proj-5ddfa986936ca339b1c5f455/sub-{}/dt-neuro-anat-t1w.tag-ACPC.id-{}/      T1.nii.gz     _info.json
- proj-5ddfa986936ca339b1c5f455/sub-{}.ses-run{}/dt-neuro-anat-t1w.tag-ACPC.id-{}/T1.nii.gz

      _info.json

### Preprocessed data

Preprocessed files are divided into 2 main categories: DW and fMRI files. The former are stored in each participant’s main folder, whereas fMRI files, if provided, are stored in folders corresponding to separate sessions.

### DW preprocessed data

Preprocessed DW data consists of two files, tagged as “preprocessed” and “clean”. The ‘preprocessed’ tag marks the data (Box 4), which has been processed online, but lacks correction for gradient nonlinearity distortions and was not aligned to T1w image (those last two steps were performed offline) - the details are described in the “Data preprocessing” section.

The “clean” tag marks the data which has been completely preprocessed and aligned to T1w image (Box 5). Consequently, the files tagged as “clean” are recommended for further analyses.

#### fMRI preprocessed data

fMRI data processing was performed both for surface and volume representations of the data, and in both cases several output files were created.

In case of surface output^92^, the output files consist of surface vertices (3D mesh), for pial and white matter, as well as inflated representation, defined for both hemispheres (Box 6a), surface data in NIfTI format containing measures at each vertices (Box 6b), surface time series data in CIFTI format (Box 6c), HTML preprocessing report (Box 6d), volumetric mask of brain (Box 6e) and confounds (nuisance regressors) representing fluctuations with a potential non-neuronal origin, identified using CompCor (Box 6f).

Volumetric preprocessing output^93^ shares a majority of files with surface preprocessing (Box 6c–f), except for files containing data in surface representation (Box 6a,b). Furthermore, two additional files are included in the volumetric input: brain mask based on BOLD image (Box 7a) and volumetric BOLD image (Box 7b).

Box 4 Organization of the preprocessed DW data files, tagged as “preprocessed”, according to the brainlife.io Data Types. proj-5ddfa986936ca339b1c5f455/sub-{}/dt-neuro-dwi.tag-raw.tag-AP.tag-normalized.tag-single_shell.tag-preprocessed.id-{}/    dwi.bvals    dwi.bvecs    dwi.nii.gz    _info.json

Box 5 Organization of the preprocessed DW data files, tagged as “clean”, according to the brainlife.io Data Types. proj-5ddfa986936ca339b1c5f455/sub-{}/dt-neuro-dwi.tag-clean.tag-ACPC.tag-normalized.tag-single_shell.id-{}/    dwi.bvals    dwi.bvecs    dwi.nii.gz    _info.json

Box 6 Organization of the fMRI preprocessed data files (surface output) according to the brainlife.io Data Types. (**a**) surface vertices (3D mesh), for pial and white matter, as well as inflated representation, defined for both hemispheres, (**b**) surface data in NIfTI format containing measures at each vertices, (**c**) surface time series data in CIFTI format, (**d**) HTML preprocessing report, (**e**) volumetric mask of brain and (**f**) confounds (nuisance regressors) representing fluctuations with a potential non-neuronal origin, identified using CompCor.

- proj-5ddfa986936ca339b1c5f455/sub-{}.ses-run{}/dt-neuro-surface-vertices.id-{}/      _info.json      left/             inflated.gii             pial.gii             white.gii      right/             inflated.gii             pial.gii             White.gii
- proj-5ddfa986936ca339b1c5f455/sub-{}.ses-run{}/dt-neuro-surface-data.id-{}/      _info.json      left.gii      Right.gii
- proj-5ddfa986936ca339b1c5f455/sub-{}.ses-run{}/dt-neuro-cifti.tag-dtseries.id-{}/      cifti.nii      _info.json
- proj-5ddfa986936ca339b1c5f455/sub-{}.ses-run{}/dt-report-html.tag-fmriprep.id-{}/      html/             sub-{}/                   figures/                           …             sub-{}.html      _info.json
- proj-5ddfa986936ca339b1c5f455/sub-{}.ses-run{}/dt-neuro-mask.tag-anat.tag-brain.id-{}/      _info.json      Mask.nii.gz
- proj-5ddfa986936ca339b1c5f455/sub-{}.ses-run{}/dt-neuro-func-regressors.id-{}/

      _info.json      regressors.json      regressors.tsv

Box 7 Organization of the additional fMRI preprocessed data files (volume output), with regard to files from surface output, according to the brainlife.io Data Types. (**a**) brain mask based on BOLD image and (**b**) volumetric BOLD image.

- proj-5ddfa986936ca339b1c5f455/sub-{}.ses-run{}/dt-neuro-mask.tag-brain.tag-bold.tag-func.id-{}/      _info.json      mask.nii.gz
- proj-5ddfa986936ca339b1c5f455/sub-{}.ses-run{}/dt-neuro-func-task.tag-raw.tag-retinotopy.tag-preprocessed.id-{}/

      bold.nii.gz      _info.json

### Data derivatives

Provided data derivatives consist of manually curated and automatically generated white matter masks, custom ROIs, T1w image segmentation, tractograms, and filtered tractograms.

### Manually curated and automatically generated masks

White matter masks manually curated in the optic chiasm region (Fig. 3b; creation described in “Data preprocessing” section) sampled to match the original T1w image resolution (Box 8):

Additional white matter mask (created from FreeSurfer segmentation of white matter), generated by brainlife.io App performing tractography^109^ (Box 9):

Box 8 Organization of the data files of manually curated optic chiasm masks according to the brainlife.io Data Types. proj-5ddfa986936ca339b1c5f455/sub-{}/dt-neuro-mask.id-{}/      _info.json      mask.nii.gz

Box 9 Organization of the data files of automatically generated optic chiasm masks according to the brainlife.io Data Types. proj-5ddfa986936ca339b1c5f455/sub-{}/dt-neuro-mask.tag-white_matter.tag-anat.id-{}/      _info.json      mask.nii.gz

### Custom ROIs

We provide a set of four masks covering the left and right optic nerve and left and right optic tract (Fig. 3e). ROIs (Box 10) are provided as individual NIfTI files containing the left and right optic tract (OT) and the left and right optic nerve (ON). Data in the files contain a ‘1’ for each voxel within the ROIs, 0 otherwise. These ROIs can be used for tracking start-end.

Box 10 Organization of the data files of custom ROIs covering optic nerves and optic tracts according to the brainlife.io Data Types. proj-5ddfa986936ca339b1c5f455/sub-{}/dt-neuro-rois/tag-aligned.id-{}/    _info.json      rois/      {}-left_ON.nii.gz      {}-left_OT.nii.gz      {}-right_ON.nii.gz      {}-right_OT.nii.gz

### T1w image segmentation

A FreeSurfer (v 7.1.1.) segmentation of T1w image, which was generated as a part of data preprocessing (see section “Data preprocessing”) and was used in tractography (Box 11a) and fMRI data preprocessing (Box 11b) is provided exclusively in brainlife.io Data Types format.

Box 11 Organization of the T1w segmentation data files (generated by the FreeSurfer) according to the brainlife.io Data Types. Data files used for (**a**) tractography and (**b**) fMRI data preprocessing purposes.

- proj-5ddfa986936ca339b1c5f455/sub-{}/dt-neuro-freesurfer.tag-ACPC.id-{}/      _info.json      output/         label/         mri/         scripts/       stats/         surf/         tmp/         touch/         trash/
- proj-5ddfa986936ca339b1c5f455/sub-{}.ses-run{}/dt-neuro-freesurfer.tag-ACPC.id-{}/

      _info.json      output/         label/         mri/         scripts/         stats/         surf/         tmp/         touch/         trash/

### Tractograms and filtering results

The results of tractography performed for the purpose of technical validation of the DW data (Box 12a) and results of its filtering with LiFE (Box 12b) are provided as part of the repository:

Box 12 Organization of the tractography data according to brainlife.io Data Types. (**a**) outcome tractogram generated for the purpose of technical validation and (**b**) results of its filtering with LiFE algorithm.

- proj-5ddfa986936ca339b1c5f455/sub-{}/dt-neuro-track-tck.tag-ensemble.id-{}/      Track.tck
- proj-5ddfa986936ca339b1c5f455/sub-{}/dt-neuro-life-tck.tag-ensemble.id-{}/

      Track.tck

### fMRI meta-files

fMRI meta-files (for a subset of 6 albinism and 4 control participants, for which fMRI source data are provided) are available on the Open Science Framework (OSF) platform: 10.17605/osf.io/XZ29Q^113^. The files are in MATLAB format.mat and provide all the necessary information for performing the retinotopy data analysis using the MrVista package (https://github.com/vistalab/vistasoft). For each participant there is a total of 6 files: description of visual stimulus presented during left and right visual hemifield stimulation (Box 13a,b, respectively), full information about the acquisition parameters, participant’s response and stimulus for left and right visual hemifield stimulation (Box 13c,d, respectively; this also includes contents of visual stimulus corresponding to given hemifield stimulation as in Box 13a,b, respectively), file containing all parameters necessary for initialization of session in MrVista (such as paths to files required in analysis; Box 13e) and mrSession file storing all information about the analysis (Box 13f):

Box 13 Organization of the fMRI meta-files stored on OSF repository. Description of visual stimulus presented during (**a**) left and (**b**) right visual hemifield stimulation, full information about the acquisition parameters, participant’s response and stimulus for (**c**) left and (**d**) right visual hemifield stimulation [this also includes contents of visual stimulus corresponding to left and right hemifield stimulation as in (**a**) and (**b**), respectively], (**e**) file containing all parameters necessary for initialization of session in MrVista (such as paths to files required in analysis) and (**f**) mrSession file storing all information about the analysis).

- {}/{}_images_left_images.mat
- {}/{}_images_right_images.mat
- {}/{}_params_left_mod.mat
- {}/{}_params_right_mod.mat
- {}/{}_mrInit_params.mat
- {}/{}_mrSession.mat

## Technical Validation

This section provides a quality assessment of the published DW and fMRI data and is based on a previously published approach^11^ comprising qualitative and quantitative measures.

### Qualitative assessment

The qualitative assessment involves (A) demonstration of the quality of alignment between anatomical, DWI, and fMRI images, and (B) demonstration of reconstruction of diffusion signal and tractography in the optic chiasm.

#### Registration of anatomical, DW, and fMRI data

A critical step in data preprocessing is to obtain the precise alignment between images of various modalities (T1w, DWI, and fMRI images; for a detailed description see Methods). The quality of registration is demonstrated by overlaying FreeSurfer’s 7.1.1 segmentation contours of white and pial matter (Fig. 2a; blue and red colors, respectively) on top of T1w image (from which they were derived; Fig. 2a, top row), DWI (Fig. 2a, middle row) and BOLD image (Fig. 2a, bottom row) for a representative participant (CON1).

#### Diffusion signal reconstruction and tractography in the optic chiasm

**Fig. 4:**
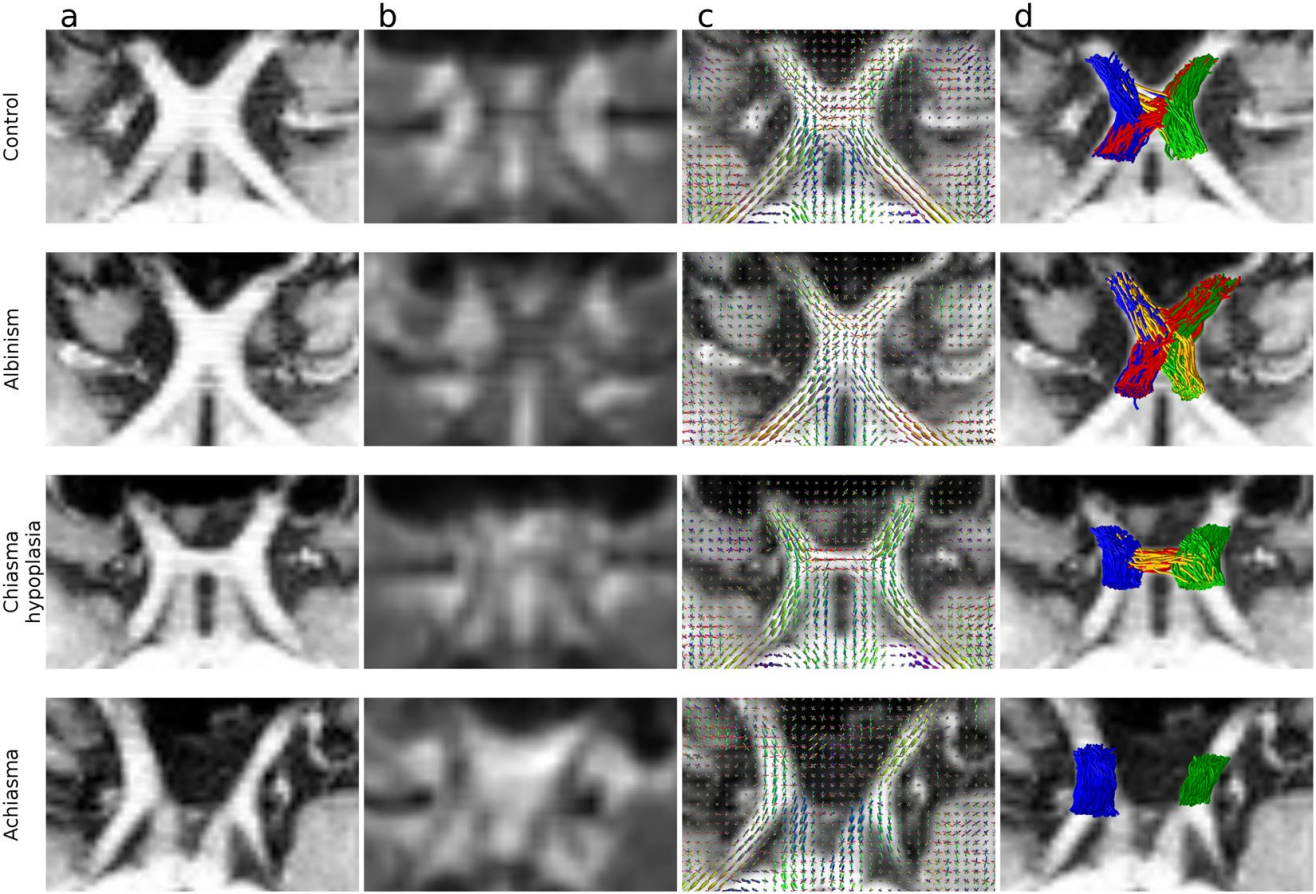
Diffusion signal reconstruction and tractography. The rows correspond to exemplary participants representing, respectively from top to bottom: control (CON1), albinism (ALB1), chiasma hypoplasia (CHP1) and achiasma (ACH1). (**a**) Pseudo-axial view of T1w image of the optic chiasm. (**b**) Pseudo-axial view of b0 image of the optic chiasm. (**c**) Pseudo-axial view of the anatomical image with overlaid estimated fiber orientation distribution function (FOD; L_max_ = 12). (**d**) Pseudo-axial view of an anatomical image with overlaid results of tractography performed between pairs of ROIs defined in Methods.

Considering the role of optic chiasm malformations as a major factor driving group differences, the quality assessment included diffusion signal modeling and tractography in this structure. Figure 4a displays representative optic chiasms in the T1w images, next to aligned dMRI b0 images (Fig. 4b). The DWI signal in each voxel was modeled using a CSD^106,114^ model in a process where an estimated single fiber response (SFR; L_max_ = 6) was used as a deconvolution kernel in the process of calculating the fiber orientation distribution function^20^ (FOD; L_max_ = 12) from acquired DWI. Figure 4c demonstrates the fit of calculated FOD in an optic chiasm region. Figure 4d demonstrates tracking results in the region of the optic chiasm. Presented results are limited only to probabilistic CSD-based tractography (iFOD2 algorithm, step size = 0.75 mm, FOD cutoff amplitude = 0.06, maximum angle between successive steps = 45°) based on already calculated ODFs (L_max_ = 12; Fig. 4c), which was done between pairs of ROIs and within manually corrected white matter mask defined in the Data derivatives paragraph of the Methods section. For the purpose of clarity, only 0.25% of the total number of generated streamlines is displayed.

### Quantitative assessment

The quantitative validation includes (A) assessment of participants’ motion during DW data acquisition, (B) SNR in raw and preprocessed DW images, (C) modeling of DW data, (D) tractography, (E) temporal SNR of fMRI data, and (F) pRF-mapping.

#### Participant’s motion during DW data acquisition

**Fig. 5:**
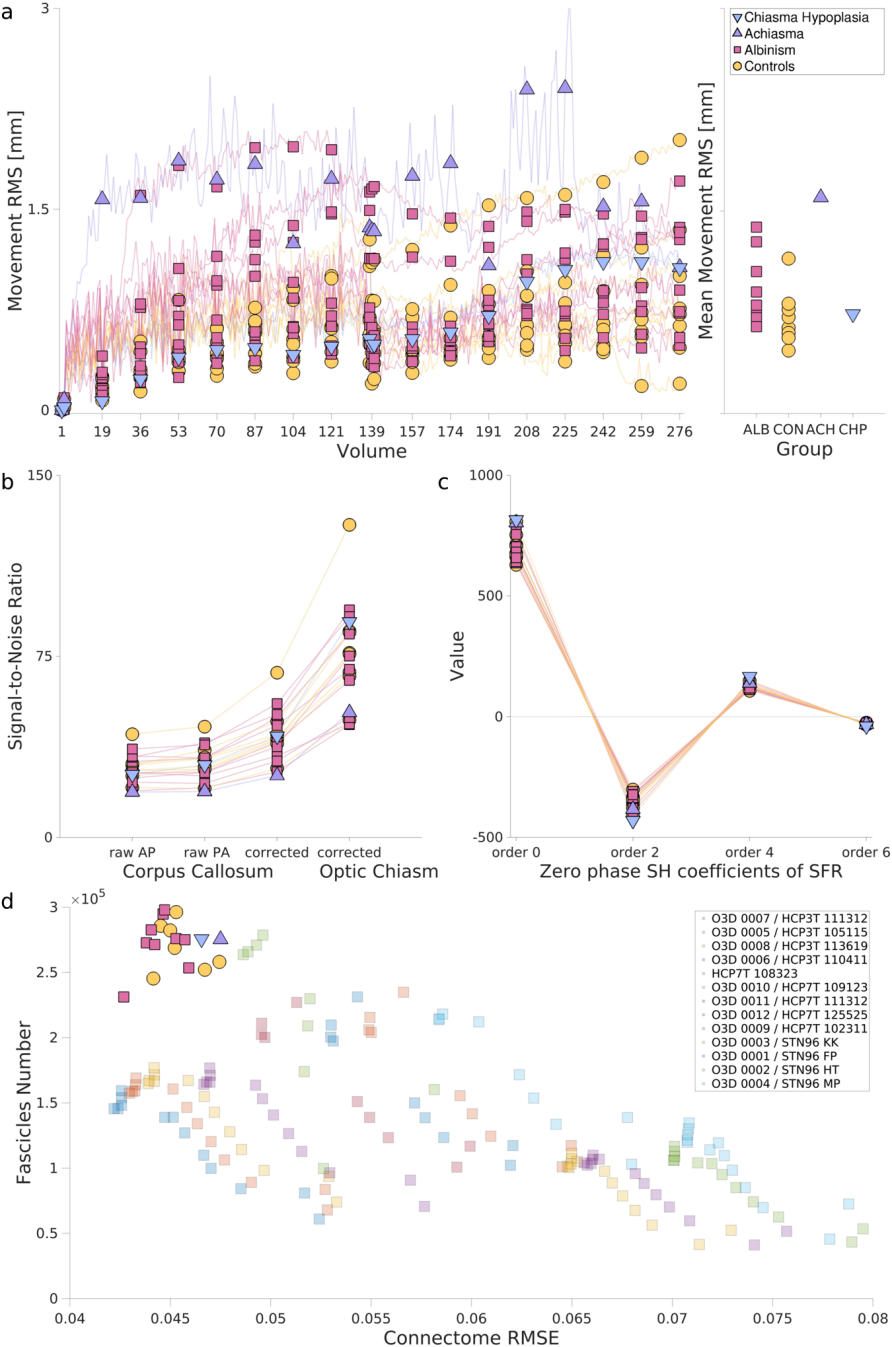
Quantitative assessment of motion, SNR, and modeling of DW data, as well as the quality of derived tractograms. (**a**) Left plot displays motion in the DW volumes (expressed as Root Mean Square of voxel displacements within brain mask, calculated with respect to the first volume) where 138 volumes with AP PED were followed by 138 volumes with opposite PED. Markers indicate the values calculated for the b0 volumes. The right-hand plot displays the mean motion RMS calculated for each participant. The color code and markers shape correspond to groups: orange circle - control, magenta square - albinism, violet up-pointing triangle - achiasma, blue down-pointing triangle - chiasma hypoplasia. (**b**) SNR in b0 images for each participant calculated from callosal voxels (selected from raw AP PED DW series, raw PA PED DW series, and corrected DWI) and optic chiasm (only for corrected DWI). (**c**) Values of zero phase (m = 0) coefficients of SFR calculated for each participant. (**d**) Results of evaluation of tractogram using LiFE method. Y-axis displays the number of fascicles remaining after filtering with LiFE, x-axis displays voxel-wise error between original signal and the one predicted from optimized tractograms. The bright markers correspond to the published datasets, while the dimmed display results for representative datasets from other publicly available DW repositories (such as 3T and 7T HCP datasets).

The participants’ motion in DWI has been calculated for concatenated AP and PA series (acquired subsequently during a single scanning session, see Methods) by calculating the RMS of each voxels’ displacement using the *Eddy* command from FSL. The displacement calculation used the first acquired voxels as a reference for all volumes and included only voxels within the brain mask. The slow, yet steadily increasing drift visible for all participants (Fig. 5a) can be well tracked with b0 images intersecting DW series, which benefited motion correction. While, the lowest RMS was observed for the control group (which can be justified by the inclusion of trained control participants, well accustomed to MRI scanning), no proof for inequality of mean displacement RMS between participants with albinism and controls was found (Student’s t-test p-value = 0.11). It should be noted that the increased motion shown by achiasmatic participant ACH1 matches the observations from the fMRI scanning session, where the data had to be discarded due to extensive motion.

The output data of *eddy* describing motion for each participant is available online on the Github repository (https://github.com/rjpuzniak/CHIASM/tree/main/Plots/Fig.5_Motion), where it is provided together with the MATLAB code of Fig. 5a.

#### SNR in DW data

In order to evaluate the quality of the DWI data, the signal-to-noise ratio (SNR) of raw and preprocessed images was measured. The computations were performed for b0 (Fig. 5b) and diffusion-weighted (along with X-, Y- and Z-axis; Suppl. Fig. 1) for corpus callosum and optic chiasm voxels. Specifically, the SNR was defined as the mean ratio of the signal in voxels (from the selected structure) to standard deviation of noise (measured in voxels outside the brain), as described in^37,115^. In the case of the corpus callosum, the SNR was calculated separately for b0 images of two raw DW series (one with AP PED and one with opposite PA PED) and for the fully preprocessed DW image (“corrected”). As expected, the comparable SNR of corrected images is increased while the preprocessing (unwarping) is performed (Fig. 5b).

Comparable analysis of SNR in optic chiasm was obstructed by the severe geometry-induced distortions present in this region, which introduce spatial warping of the chiasm. Although theoretically this problem can be addressed by using new sets of optic chiasm masks (created separately for images warped in AP and PA directions), drawing new masks on top of DW images in heavily distorted regions is practically extremely challenging and is highly likely to introduce inaccuracies. Instead, the SNR was calculated for the fully preprocessed (corrected) DWI images, where the optic chiasm mask was cropped from a manually curated white matter mask (Fig. 5b). The observed higher SNR in optic chiasm (compared to corpus callosum) could be due to multiple reasons that were not tested by the authors. We speculate that it might be the result of using a 64-channel Radio Frequency coil, which measures stronger signals from peripheral brain regions in comparison to deeper regions such as the corpus callosum. The brainlife.io application implementing the SNR computation in the corpus callosum can be found at^116^ which follows the outlined SNR calculation strategy presented in^37,115^.

#### Coefficients of the single fiber response (SFR) function

The quality of modeling of DWI was assessed by plotting the zero phase coefficient of the SFR function (calculated for L_max_ = 6 with *dwi2response* command from MRtrix, which used a iterative algorithm for SFR voxels selection and response function estimation;^107^ Fig. 5c). The observed plot is in agreement with theoretical expectations, where successive non-zero (even) terms are of opposite signs and of decreasing absolute value.

#### Quality of tractography

Finally, the quality of the created tractogram has been assessed with the LiFE algorithm (see Diffusion signal reconstruction, tractography and statistical evaluation in Methods). Figure 5d demonstrates the correlation between the number of fascicles of non-zero weights (y-axis) and Connectome Root Mean Square Error (RMSE;^9,103^). The data points for all CHIASM participants demonstrate a high number of weighted fascicles (positively contributing to measured signal, y-axis on Fig. 5d) combined with a low connectome’s RMSE (measuring the discrepancy between signal predicted from weighted fascicles and measured signal, y-axis). Those measures replicate previous findings of high-quality diffusion scans (HCP/O3D^11,117^).

#### tSNR of fMRI data

**Fig. 6:**
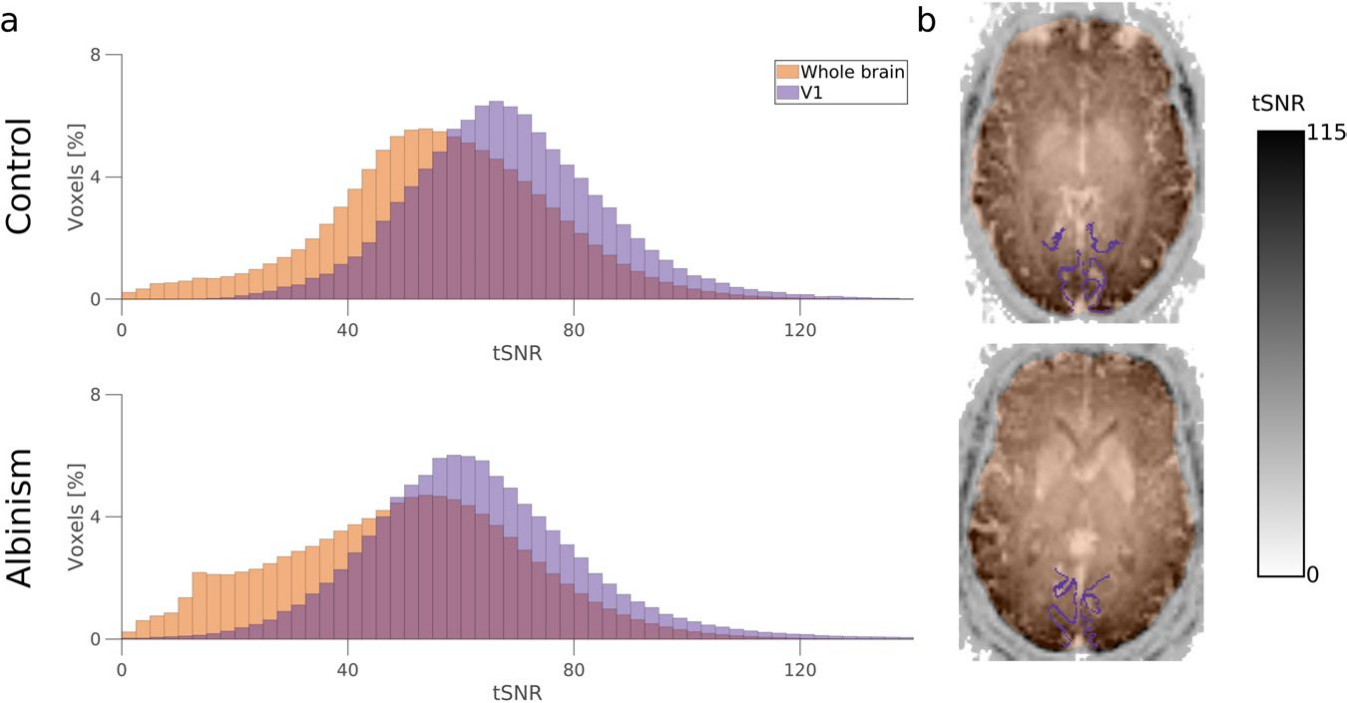
BOLD image temporal SNR in V1 and whole-brain volume. (**a**) Histograms of tSNR calculated in whole brain volume (orange) and V1 region (defined according to Benson’s atlas; violet). (**b**) Whole brain (orange) and V1 (violet) masks overlaid on maps of tSNR calculated for representative participants (top - control participant CON1, bottom - participant with albinism ALB1).

The quality of fMRI images was assessed using a temporal SNR (tSNR) measure (Fig. 6). Specifically, the tSNR in the BOLD images was calculated for two areas - whole brain volume (derived from BOLD images with *fMRIPrep - Volume Output*^93^ App) and primary visual cortex (V1) mask derived from T1w image using Benson’s atlas^118–120^. The tSNR has been calculated using the code provided in^121^ (https://github.com/psychoinformatics-de/studyforrest-data-aligned). The mean values of tSNR calculated for participants with albinism and controls (51.0 and 57.7, respectively) correspond well to the tSNR previously reported for voxel volumes of 16.625 mm^3^ ^122^.

#### Population Receptive Fields (pRF) Mapping

The pRF-mapping data derivatives and methods were described in a previous publication^51^. Briefly: The pRF sizes and positions can be estimated from the fMRI data and visual stimulus position time course. The BOLD response of each voxel can be predicted using a circular 2D-Gaussian model of the neuronal populations receptive field defined by three stimulus-referred parameters i.e. x0, y0, σ where x0 and y0 are the coordinates of the receptive field center and σ is it’s spread^61^. The predicted BOLD signal can be calculated by convolution of the stimulus sequence for the respective pRF-model and its three parameters with the canonical hemodynamic response function^123^. Based on this, the optimal pRF parameters can be found by minimizing the residual sum of squared errors (RSS) between the predicted and observed BOLD time-course. Only voxels will be retained whose explained variance exceeded a threshold of 15%.

## Usage Notes

This data is organized according to BIDS standard^112^, whenever applicable (additionally, in all cases the data is organized according to brainlife.io Data Types), and are stored in documented standard NIfTI format. The data is to be accessed at the brainlife.io computing platform either by (A) the web interface of brainlife.io and/or (B) a command-line interface (https://github.com/brainlife/cli). CLI offers means to query and download partial or full data. This utility is further expanded when using a web interface, which in addition to selection and download of data allows for online processing with provided brainlife.io Apps Table 5.

**Table 5:** Code implementing each processing step.

Type	Preprocessing step	URL to code
T1w	1. DICOM conversion	—
	2. Anonymization	—
	3. ACPC Alignment	—
	4. Tissue Segmentation	—
	5. Tissue segmentation	https://github.com/brainlife/app-freesurfer/tree/7.1.1
DWI	1. DICOM conversion	https://github.com/rjpuzniak/CHIASM/blob/main/Uploading_and_Preprocessing/Diffusion_and_T1-weighted_data/1_Upload_raw_DW_T1w.sh
	2. Denoising	https://github.com/brain-life/app-mrtrix3-preproc
	3. Removal of Gibbs ringing	https://github.com/brain-life/app-mrtrix3-preproc
	4. Eddy currents distortions corrections	https://github.com/brain-life/app-mrtrix3-preproc
	5. Geometrical distortions corrections	https://github.com/brain-life/app-mrtrix3-preproc
	6. Correction for head motion	https://github.com/brain-life/app-mrtrix3-preproc
	7. Correction for bias field	https://github.com/brain-life/app-mrtrix3-preproc
	8. Correction for gradient nonlinearities	https://github.com/rjpuzniak/CHIASM/blob/main/Uploading_and_Preprocessing/Diffusion_and_T1-weighted_data/2_Apply_GNC.sh
	9. Coregistration to T1w image & Rotation of b-vectors	https://github.com/rjpuzniak/CHIASM/blob/main/Uploading_and_Preprocessing/Diffusion_and_T1-weighted_data/3_Align_DW_to_T1w.sh
fMRI	1. DICOM conversion	https://github.com/rjpuzniak/CHIASM/blob/main/Uploading_and_Preprocessing/fMRI/1_Extract_anonymize_upload_fMRI_data.sh
	2. Geometrical distortions corrections	https://github.com/brainlife/app-fmriprep/tree/20.2.1
	3. Registration to T1w image	https://github.com/brainlife/app-fmriprep/tree/20.2.1
	4. Slice-time correction	https://github.com/brainlife/app-fmriprep/tree/20.2.1
	5. Motion correction	https://github.com/brainlife/app-fmriprep/tree/20.2.1
	6. Removal of physiological noise	https://github.com/brainlife/app-fmriprep/tree/20.2.1
Masks and ROIs	1. Alignment of the custom mask to T1w image	https://github.com/rjpuzniak/CHIASM/blob/main/Uploading_and_Preprocessing/ROIs_and_Mask/1_Align_anonymize_upload_ROIs.sh
	2. Alignment of the ROIs to T1w image	https://github.com/rjpuzniak/CHIASM/blob/main/Uploading_and_Preprocessing/ROIs_and_Mask/1_Align_anonymize_upload_ROIs.sh
	3. Removal of interpolation artifacts	https://github.com/rjpuzniak/CHIASM/blob/main/Uploading_and_Preprocessing/ROIs_and_Mask/1_Align_anonymize_upload_ROIs.sh

Code implementing the processing pipeline is available on https://github.com/rjpuzniak/CHIASM.

## Supplementary information

Supplementary Information

## Data Availability

The processing was performed mainly using brainlife.io services (https://brainlife.io/apps), which together with the code are available online. The offline preprocessing was performed with freely accessible neuroimaging tools. The preprocessing steps, together with the references to the Software/Apps are provided separately for the T1w, DW, fMRI data, and hand-curated ROIs and mask (Tables 1–4, respectively). The web links to source code are provided separately in the Table 5.
