# Supplementary material for: CHIASM, the human brain albinism and achiasma MRI dataset
Source: Sci Data. 2021 Nov 26;8:308. doi: 10.1038/s41597-021-01080-w (PMC8626506; doi:10.1038/s41597-021-01080-w)
Supplement: Supplementary file 1 — Supplementary Information [file 41597_2021_1080_MOESM1_ESM.docx]

**CHIASM, the human brain albinism and achiasma MRI dataset**

Robert J. Puzniak^1^, Brent McPherson^2+^, Khazar Ahmadi^1+^, Anne Herbik^1^, Jörn Kaufmann^3^, Thomas Liebe^4^, Andre Gouws^5^, Antony B. Morland^6^, Irene Gottlob^7^, Michael B. Hoffmann^1,8#^, Franco Pestilli^9#^*

We describe a collection of T1-, diffusion- and functional T2*-weighted magnetic resonance imaging data from human individuals with albinism and achiasma. This repository can be used as a test-bed to develop and validate tractography methods like diffusion-signal modeling and fiber tracking as well as to investigate the properties of the human visual system in individuals with congenital abnormalities. The MRI data is provided together with tools and files allowing for its preprocessing and analysis, along with the data derivatives such as manually curated masks and regions of interest for performing tractography.

## **Affiliations**

^1^ Visual Processing Lab, Department of Ophthalmology, Otto-von-Guericke-University, Leipziger-Str. 44 (H. 60B), 39120, Magdeburg, Germany.

^2^ Pestilli Lab, Department of Psychological and Brain Sciences, Program in Cognitive Science, Indiana University Bloomington, 1101 E 10th Street, Bloomington, Indiana, 47405, USA.

^3^ Department of Neurology, Otto-von-Guericke-University, Leipziger-Str. 44 (H. 60A/60B), 39120 Magdeburg, Germany.

^4^ Department of Psychiatry and Psychotherapy, Jena University Hospital, Philosophenweg 3, 07742, Jena, Germany.

^5^ York Neuroimaging Centre, Department of Psychology, University of York, York, YO10 5DD, United Kingdom.

^6^ Centre for Neuroscience, Hull-York Medical School, Heslington, York, YO10 5DD, United Kingdom.

^7^ Department of Neuroscience, Psychology & Behaviour, University of Leicester, University Road, Leicester, LE1 7RH, United Kingdom.

^8^ Center for Behavioral Brain Sciences, Otto-von-Guericke-Universität, Universitätsplatz 2 (G24-205), 39106, Magdeburg, Germany.

^9^ Pestilli Lab, Department of Psychological and Brain Sciences, Engineering, Computer Science, Programs in Neuroscience and Cognitive Science, School of Optometry, and Indiana Network Science Institute, Indiana University Bloomington, 1101 E 10th Street, Bloomington, Indiana, 47405, USA.

^+,#^ These authors contributed equally

* Correspondence and requests for materials should be addressed to F.P. (email: [pestilli@utexas.edu](mailto:pestilli@utexas.edu))

| **Table of contents of the supplementary file** | | | |
| --- | --- | --- | --- |
| **Content** | **Description** | | **Page** |
| Supplementary Table 1. | Ophthalmological information about patients with albinism taking part in fMRI acquisition. | | 2 |
| Supplementary Table 2. | Information about controls taking part in fMRI acquisition. | | 3 |
| Supplementary Figure 1. | SNR of DW data in corpus callosum and optic chiasm. | | 4 |
| Supplementary Box 1. | Open Access Data Use Agreement | | 5 |
| Supplementary Box 2. | Restricted Data Use Agreement | | 6-7 |

| **brainlife-**  **ID** | **Stimulated (Dominant) Eye** | **Decimal**  **VA**^1^ | | **Fixation stability**  **[%]** ^2^ | **Optic nerve**  **miss-routing**  **extent [°]**  **(fMRI-**  **based)** ^3^ | **Visually evoked potentials**  **(correlation coefficient)** ^4^ | | | **Ocular and pigmentation characteristics in albinism** ^5^ | | |
| --- | --- | --- | --- | --- | --- | --- | --- | --- | --- | --- | --- |
|  |  | Eye | |  |  | check size [°] | | | ocular characteristics ^6^ | | |
|  |  | *L* | *R* |  |  | *0.5* | *1.0* | *2.0* | *iris*  *trans-*  *lucency ^7^* | *Fundus hypopigmentation & foveal hypoplasia ^8^* | *optic*  *nerve*  *head ^9^* |
| ALB1^a^ | Left | **0.10** | 0.10 | 65 | 9.34 | -0.86 | -0.89 | -0.76 | 1 | 3 | 1 |
| ALB2 | Right | 0.05 | **0.06** | 99 | - | -0.86 | -0.82 | -0.56 | 4 | 1 | 2 |
| ALB3 | Left | **0.13** | 0.13 | - | - | -0.89 | -0.95 | -0.85 | 4 | 4 | 3 |
| ALB4^α^ | Right | 0.25 | **0.32** | - | - | -0.86* | -0.81* | -0.80* | - | - | - |
| ALB5^e^ | Left | **0.40** | 0.32 | 95 | 3.09 | -0.89 | -0.96 | -0.94 | 1 | 3 | 0 |
| ALB6 | Left | **0.50** | 0.25 | - | 4.20 | -0.85 | -0.70 | -0.33 | 3 | 1 | 2 |
| ALB7^c^ | Left | **0.16** | 0.10 | 79 | 8.29 | -0.95 | -0.96 | -0.95 | 4 | 1 | 2 |
| ALB8^b^ | Left | **0.16** | 0.13 | 83 | 9.33 | -0.75 | -0.72 | -0.49 | 3 | 1 | 2 |
| ALB9^d^ | Left | **0.32** | 0.14 | - | 5.22 | -0.87 | -0.95 | -0.67 | 3 | 1 | 0 |
| CHP1^β^ | Right | 0.25 | **0.63** | - | - ^†^ | - | - | - |  | | |
| ACH1 | Left | **0.20** | 0.10 | 84 | full^‡^ | -0.48 | -0.84 | -0.87 |  |  |  |

**Supplementary Table 1. Ophthalmological information about patients with albinism taking part in fMRI acquisition.**

^a,b,c,d,e^ participated in study by [^1^](https://www.zotero.org/google-docs/?ppBHJH) as A1, A2, A4, A5, A6, respectively.

^α,β^ data accessible upon request (non-open policy).

^1^ far visual acuity; bold – stimulated (dominant) eye; L – left; R – right eye.

^2^ fixation stability (stimulated eye) within the 3º window as detailed in [^1^](https://www.zotero.org/google-docs/?P6hFIr).

^3^ as detailed in [^1^](https://www.zotero.org/google-docs/?LQiWb3).

^4^ interocular correlation of VEP-based interhemispheric activation differences as detailed in [^2^](https://www.zotero.org/google-docs/?gayzOp) at 98% contrast or *****20% contrast.

^5^ according to conventions detailed in [^3^](https://www.zotero.org/google-docs/?blxLmY).

^6^ identical scores for both eyes.

^7^ score range: 1 (peripheral punctate iris translucency) to 4 (complete translucency, including the pupillary margin).

^8^ score range: 1 (peripheral retinal hypopigmentation, foveal structures visible) to 4 (pronounced peripheral & central hypopigmentation, foveal & macular hypoplasia, atypical choroidal vessels crossing presumed

macular region).

^9^ score range: 0 (non-pathologic optic nerve head) to 4 (dysplasia of optic nerve head).

^†^ detailed in the specific case study by [^4^](https://www.zotero.org/google-docs/?gI0OVN).

^‡^ complete absence of optic chiasm resulting in complete absence of crossing fibres, i.e. full extent of misrouting.

| **BrainLife ID** | **Stimulated eye during fMRI** |
| --- | --- |
| CON1 | Right |
| CON2 | Right |
| CON3 | Left |
| CON8 | Right |

**Supplementary Table 2. Information about controls taking part in fMRI acquisition.**


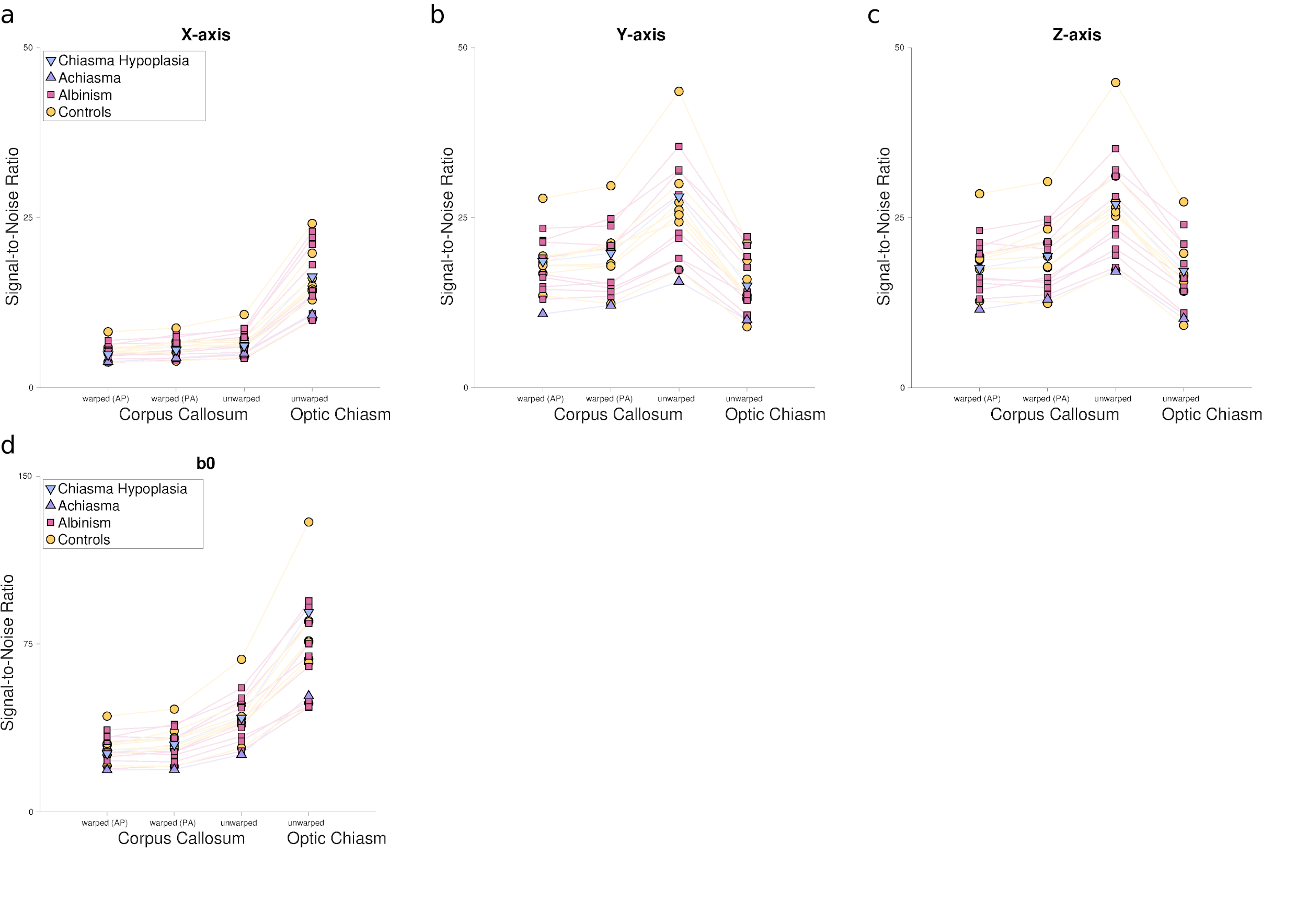


**Supplementary Figure 1. SNR of DW data in corpus callosum and optic chiasm.** SNR for each participant calculated from callosal voxels (selected from: raw AP DWI series, raw PA DWI series, and corrected DWI) and optic chiasm (only for corrected DWI). The SNR is reported for diffusion weighting along (**a**) x-axis, (**b**) y-axis, (**c**) z-axis, where axes are defined with regard to AC-PC space. Figure (**d**) displays SNR in absence of diffusion weighting (b0 image).

| **CHIASM Open Access Data Use Agreement**  I request access to data of control participants from the “CHIASM, the human brain albinism and achiasma MRI dataset” (further referred to as “CHIASM”) repository for the purpose of scientific investigation, teaching or the planning of clinical research studies and agree to the following terms:   1. I will receive access to de-identified data and will not attempt to establish the identity of, or attempt to contact any of the CHIASM dataset repository study participants. 2. I will not redistribute these data beyond the uses outlined in this agreement and my data use application. 3. I will require anyone in my team who utilizes these data to comply with this data use agreement. 4. I will provide a list of all the persons who will use these data and the analyses that are planned using these data by emailing Michael Hoffmann <michael.hoffmann@med.ovgu.de>. 5. Before granting access to the data to other team members I will provide an updated list of users by emailing Michael Hoffmann <michael.hoffmann@med.ovgu.de>. 6. I will comply with any rules and regulations imposed by my institution, local and federal government, and institutional review board. 7. I will ensure that investigators who utilize the CHIASM dataset use appropriate administrative, physical and technical safeguards to prevent use or disclosure of the data other than as provided for by this agreement as mandated by local, federal and institutional regulations and laws. 8. I will report any use or disclosure of the data not described by this agreement of which I become aware within 15 days of becoming aware of such use or disclosure. 9. The data originators request no authorship to any of the publications generated using the CHIASM dataset. 10. Any publication I will produce (abstracts or articles) using data from the CHIASM dataset will cite the Data Descriptor of the CHIASM dataset as the source of data and will acknowledge the CHIASM dataset funding sources. The citation to be used for abstracts and articles and the text to be added to the Acknowledgements section are provided below:   *Text to be added in the Methods section with the citations:*  "Data used in the preparation of this work were obtained from the CHIASM dataset available at brainlife: https://doi.org/10.25663/brainlife.pub.9, (Puzniak et al., 2021, Avesani et al., 2019).  Citations:  <Puzniak et al. To be added at Proof stage.>  Avesani, P., McPherson, B., Hayashi, S. et al. The open diffusion data derivatives, brain data upcycling via integrated publishing of derivatives and reproducible open cloud services. Sci Data 6, 69 (2019). https://doi.org/10.1038/s41597-019-0073-y  *Text to be added to the Acknowledgments:*  "Data collection and sharing for this project was provided by the CHIASM dataset available at brainlife.io. The brainlife.io project was supported by NSF IIS 1636893, NSF IIS 1912270, NIH NIBIB 1R01EB029272, NSF BCS 1734853 and a Microsoft Investigator Fellowship."  I understand that failure to abide by these guidelines will result in termination of my privileges to access the CHIASM repository data and potential legal liabilities. |
| --- |

**Supplementary Box 1. Open Access Data Use Agreement.**

| **CHIASM Restricted Data Use Agreement**  The following Data Use Terms must be signed, submitted and approved for investigators to receive access to Restricted Data of the “CHIASM, the human brain albinism and achiasma MRI dataset” (further referred to as “CHIASM”) repository. Given the uniqueness of patients’ condition, these special precautions are essential in order to protect the privacy of our subjects and to prevent any inappropriate disclosure of subject identity.  **A. Definitions of Restricted Data Elements covered by these terms:**   1. **MRI Data.** This category covers T1-, diffusion- and BOLD-weighted images acquired from the non-control participants of the CHIASM repository, and any derivative data generated from those images.   **B. Who Can Obtain Access:**  Investigators are expected to meet one of the following criteria to be qualified to receive access to CHIASM Restricted Data:   1. You are a Principal Investigator (PI) of scientific research at a university, a research organization (including commercial entities) or a government agency who is the leader of a laboratory or research team or who is working independently; or 2. You provide the name of the PI who is overseeing your research and is approved for access under #1. 3. If you do not meet either of the above criteria you may be considered qualified based on a track record of scientific publications or on the basis of a written reference from someone who meets qualification #1, verifying that the data will be used only for the purpose of legitimate scientific research. | | |
| --- | --- | --- |
|  |  |  |
| **C. Obligations of Investigators**  I request access to restricted data belonging to the CHIASM repository. IN ADDITION TO the rules I have accepted in the Open Access Data Use Terms, to protect the privacy of CHIASM participants I agree to abide by the following terms. | | **Applicant**  **Initials** |
|  |  |  |
| 1. | - I am a Principal Investigator (PI) as defined above in B.1   **OR**   - I am not a PI. Please complete and submit this application.We must receive an application from your PI before we can process your application.   Name of PI (please print legibly) _______________________________ |  |
| 2. | **I will not redistribute or share Restricted Data** with others, including individuals in my team, unless they have independently applied and been granted access to the Restricted Data of the CHIASM repository. |  |
| 3. | I will **keep the Restricted Data secure (password protected**, such that the data are only accessible to individuals who have already been granted access). |  |
| 4. | I will comply with all relevant rules and regulations imposed by my institution as well as my local and federal government. This may mean that I need my research to be approved by a committee that oversees research on human subjects, e.g. my IRB or Ethics Committee. Different committees operate under different national, state and local laws and may interpret regulations differently, so it is important to ask about this. If needed and upon request, the certificate stating that you have accepted the CHIASM Restricted Data Use Agreement will be provided. |  |
| 5. | I affirm that I will use the Restricted Data exclusively for the purpose of scientific research, technology development, or education under the auspices of an academic, research, government or commercial entity. |  |
| 6. | I agree to provide additional specific justification if requested before being granted access to any Restricted Data Elements.. |  |
| 7. | I also understand and have accepted the provisions in the CHIASM Open Access Data Use Agreement, as provided in the “CHIASM, the human brain albinism and achiasma MRI set” publication’s supplementary information. |  |
| 8. | I agree to delete specific CHIASM datasets if requested to do so by Michael B. Hoffmann, e.g., if certain released datasets are found to have identifying information inadvertently included. |  |
| 9. | I understand that failure to abide by these rules may result in termination of my privileges to access CHIASM data. In addition, inappropriate use, redistribution or publication of restricted access data, including any actions that may violate the privacy of CHIASM participants, may lead to CHIASM authors taking other actions against me, including informing my institution, relevant journal(s), and relevant funding agencies. |  |
| Applicant Information (required for all applicants):  Name: _________________________________________________________  Appointment or title (e.g. Professor, Postdoc, Graduate Student):______________________________________________  Institutional affiliation: _____________________________________________  Highest academic degree: _________________________________________  Email address: __________________________________________________  Daytime telephone number: ________________________________________  **For PIs only**: Names of non-PI applicants in your group who have applied or will be applying for access, to the extent known. All users of CHIASM Restricted Data **MUST** apply independently for access.  ____________________________________________________________________  ____________________________________________________________________  Applicant Signature: ________________________________________  Date: ________________________________________  **Note:** Applications must be filled by hand and sent as scanned pdf file to michael.hoffmann@med.ovgu.de | | |

**Supplementary Box 2. Restricted Data Use Agreement**

**Bibliography**

[1. Ahmadi, K. *et al.* Population receptive field and connectivity properties of the early visual cortex in human albinism. *NeuroImage* **202**, 116105 (2019).](https://www.zotero.org/google-docs/?vppP7W)

[2. Hoffmann, M. B. *et al.* Visual Pathways in Humans With Ephrin-B1 Deficiency Associated With the Cranio-Fronto-Nasal Syndrome. *Invest. Ophthalmol. Vis. Sci.* 56, 7427–7437 (2015).](https://www.zotero.org/google-docs/?vppP7W)

[3. Schmitz, B. *et al.* Configuration of the Optic Chiasm in Humans with Albinism as Revealed by Magnetic Resonance Imaging. *Invest. Ophthalmol. Vis. Sci.* 44, 16–21 (2003).](https://www.zotero.org/google-docs/?vppP7W)

[4. Ahmadi, K. *et al.* Triple visual hemifield maps in a case of optic chiasm hypoplasia. *NeuroImage* 215, 116822 (2020).](https://www.zotero.org/google-docs/?vppP7W)
